# Biocompatibility testing and antioxidant properties of cerium dioxide nanoparticles in human nervous system cells

**DOI:** 10.1007/s00204-025-04096-y

**Published:** 2025-06-06

**Authors:** Natalia Fernández-Bertólez, Assia Touzani, Lucía Ramos-Pan, Ana Teresa Reis, João Paulo Teixeira, Blanca Laffon, Vanessa Valdiglesias

**Affiliations:** 1 Universidade da Coruña, NanoToxGen Group, CICA–Centro Interdisciplinar de Química e Bioloxía, Department of Biology, 15071 A Coruña, Spain; 2 Instituto de Investigación Biomédica de A Coruña (INIBIC), 15006 A Coruña, Spain; 3 Environmental Health Department, National Institute of Health, 4050-600 Porto, Portugal; 4 EPIUnit-Instituto de Saúde Pública, Universidade Do Porto, 4050-600 Porto, Portugal; 5 Laboratório Para a Investigação Integrativa E Translacional Em Saúde Populacional (ITR), 4050-600 Porto, Portugal; 6 Universidade da Coruña, DICOMOSA Group, CICA–Centro Interdisciplinar de Química e Bioloxía, Department of Psychology, 15071 A Coruña, Spain

**Keywords:** Cerium dioxide nanoparticles, Neuronal cells, Glial cells, Cytotoxicity, Oxidative stress, Antioxidant capacity

## Abstract

Cerium dioxide nanoparticles (CeO_2_ NP), or nanoceria, are versatile materials with interesting properties for industry and medicine fields, particularly redox properties and catalytic activity. Because of their distinctive features, they have gained high attention in biomedical and pharmacological research to be employed in drug delivery, tissue regeneration, radioprotection, or diagnostic imaging. However, previous works reported that nanoceria may also induce reactive oxygen species (ROS) under certain conditions, leading to cellular stress, cellular damage, or cell death. In this study, the effects of CeO_2_ NP on cell viability and morphology as well as their influence on oxidative stress (both oxidant and ROS scavenging capacities) were investigated in nervous system cells (SH-SY5Y neuronal and A172 glial cells) treated with a wide range of CeO_2_ NP concentrations (1–100 µg/mL) for several treatment times. Results obtained showed that, despite being stable in time and effectively internalized by both cell types, CeO_2_ NP did not produce significant decrease in viability, evaluated by MTT assay, morphological alterations, or intrinsic cell-free ROS, but they generated cellular ROS limited to longer exposure periods. Furthermore, CeO_2_ NP demonstrated a certain intrinsic ability to scavenge ROS generated by H_2_O_2_ in both tested cell types, more pronounced in neuronal cells. These results confirm the good biocompatibility of nanoceria on human nervous system cells and support further exploring their potential use in biomedicine field, particularly for those therapeutic and diagnostic applications related to the nervous system.

## Introduction

Cerium dioxide nanoparticles (CeO_2_ NP), also known as nanoceria, are versatile materials that have gained attention in biological, biomedical and pharmacological research due to their unique properties (Casals et al. 2020; Saifi et al. 2021). Some of their potential applications, such as drug delivery, tissue regeneration, radioprotection, or diagnostic imaging, are based on their distinctive features (reviewed in (Thakur et al. 2019)). These physical and chemical characteristics, including size and shape, crystal structure, redox properties, catalytic activity, and surface chemistry, contribute to their diverse raising applications in medicine, but also in catalysis, and materials science, among others (Scirè and Palmisano 2020).

At nanoscale, the cerium ability to switch between the + 3 and the + 4 oxidation states enables these NP to act as antioxidants driven by the release/store of oxygen atoms within the crystal lattice generating oxygen vacancies (defect sites) on the NP surface, which is crucial in scavenging free radicals and mitigating oxidative stress. Moreover, CeO_2_ NP exhibits notable catalytic activity, making them useful in various catalytic reactions due to their oxygen vacancies and redox cycling abilities (Nelson et al. 2016); they exhibit enzyme-like catalytic properties, such as superoxide dismutase (SOD) and catalase activities, so they can mimic their functions, involved in neutralizing harmful reactive species and promoting cellular defense mechanisms against oxidative damage, contributing to their therapeutic potential (Naz et al. 2017; Rzigalinski et al. 2017).

**Fig. 1:**
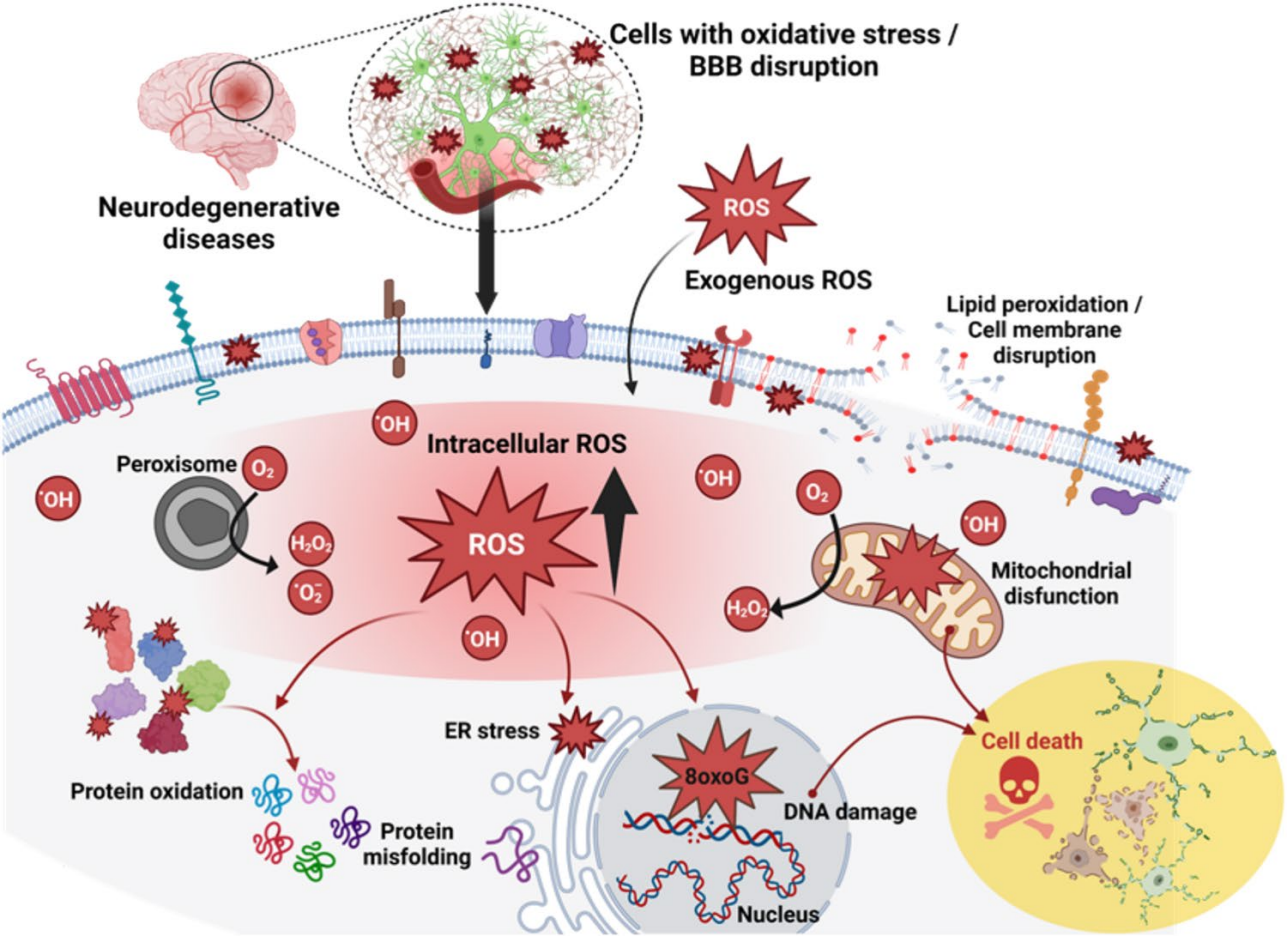
Role of oxidative stress in neurodegenerative disorders. Production of reactive oxygen species (ROS) is a natural cellular process that occurs mainly as a byproduct of cellular metabolism, particularly within peroxisomes or the mitochondrial electron transport chain, but can also arise from external factors like pollution, radiation, cigarette smoke, and pesticides. Once the production of free radicals, such as superoxide (^•^O_2_^−^), hydrogen peroxide (H_2_O_2_), or hydroxyl radicals (^•^OH), overwhelm the cellular antioxidant mechanisms, their excessive accumulation can lead to detrimental effects compromising cellular function. Potential consequences include lipid peroxidation, causing cell membrane disruption, protein oxidation/aggregation and the buildup of misfolded proteins causing endoplasmic reticulum stress, mitochondrial dysfunction increasing ROS production, and oxidative DNA damage (primarily 8-oxo-7,8-dihydroguanine (8-oxoG)). Neurons in the central nervous system are especially susceptible to oxidative stress due to their high energy demands, the abundance of polyunsaturated lipids in their membranes, and their limited antioxidant capacity. Therefore, oxidative stress can lead to mitochondrial impairment, altered protein structures and functions, DNA mutations and genetic instability, disruptions in cellular signaling and in synaptic transmission, and chronic neuroinflammation, that trigger programmed cell death mechanisms and accelerates neurodegeneration. Created in Biorender by Fernández-Bertólez N. in 2024 https://BioRender.com/h89v100

The antioxidant capacity and the enzyme-like activity of CeO_2_ NP contribute significantly to their potential usefulness in the diagnosis and treatment of diseases and tumors of the human central nervous system (CNS) (Saifi et al. 2021; Sundararajan et al. 2021). Neurodegenerative diseases, such as Alzheimer's and Parkinson’s, are associated with oxidative stress and the accumulation of reactive oxygen species (ROS). Furthermore, oxidative stress often triggers inflammatory responses in the CNS, contributing to the progression of neurodegenerative disorders (Fig. 1) (Rzigalinski et al. 2017; Nele et al. 2023). CeO_2_ NP behavior aids in reducing oxidative stress by scavenging ROS, mitigating inflammation by modulating the immune response, and protecting cellular components, potentially slowing down the progression of these diseases (Naz et al. 2017; Shcherbakov et al. 2019). In the case of CNS tumors, the enzyme-like activity of nanoceria can offer radioprotection to healthy tissues surrounding the tumor site during radiation therapy, helping minimize collateral damage (Naz et al. 2017; Shcherbakov et al. 2019). In addition to their antioxidant ability, their optical properties make CeO_2_ NP suitable as contrast agents for imaging techniques like fluorescence imaging or magnetic resonance imaging, enhancing their accuracy in detecting specific biomarkers or pathological changes associated with neurological disorders (Sack-Zschauer et al. 2017).

Neuroblastoma SH-SY5Y cells and glioblastoma A172 cells are two cell lines often employed as model cultures for human nervous system. Their respective characteristics make them valuable tools for in vitro toxicology and pharmacology studies focusing on neuronal- and glial-related research, aiding in the development of potential therapeutics and understanding disease mechanisms (Laffon et al. 2017; Fernández-Bertólez et al. 2018; Lopez-Suarez et al. 2022).

Studies addressing the toxic effects of CeO_2_ NP in human cells so far display diverse outcomes. On the one hand, as previously indicated, CeO_2_ NP exhibits promising antioxidant capabilities and anti-inflammatory traits, scavenging ROS, reducing inflammation, and protecting cells from oxidative damage (Goujon et al. 2021). On the other hand, they might generate ROS under certain conditions, leading to cellular stress (Forest et al. 2017; Gliga et al. 2017). CeO_2_ NP induced ROS-mediated cytotoxic effects at high concentrations and prolonged exposure periods, causing cellular damage, apoptosis, or cell death (Casals et al. 2020). This complex interplay between beneficial effects and potential risks underscores the need for a thorough understanding of the safety profile and mode of action of these NP. Understanding the intricate balance between therapeutic effects and the potential toxicity is essential for their safe and effective application in biomedical contexts (Heckman et al. 2020). Further research is necessary to comprehensively evaluate their cellular and genetic effects, optimizing their beneficial use while minimizing potential risks, especially within the complex CNS environment.

The aim of this study was to determine the effects of CeO_2_ NP on cell viability and morphology, and to investigate their influence on oxidative stress (both oxidant and ROS scavenging capacities) in nervous system cells (SH-SY5Y neuronal and A172 glial cells). To that end, after determining their physical–chemical characteristics and their internalization by both cell types, cellular effects were assessed by MTT assay and morphological analysis. Their ability to generate ROS in acellular and cellular environments and induce oxidative DNA damage was evaluated by 2′,7′-dichlorodihydrofluorescein diacetate (DFDH-DA) and formamidopyrimidine DNA glycosylase (fpg)-modified comet assays, respectively. Additionally, their competence for intracellular ROS scavenging was also explored.

## Materials and methods

### Chemicals

Cerium (IV) oxide nanoparticles (CAS No. 1306-38-3), MTT (CAS No. 298-93-1), dimethyl sulfoxide (DMSO) (CAS No. 67-68-5) ACS reagent ≥ 99.9%, Triton X-100 (CAS No. 9002-93-1), potassium bromate (KBrO_3_) (CAS No. 7758-01-2) for analysis EMSURE^®^ Reag. Ph Eur, 2′,7′-dichlorodihydrofluorescein diacetate (DCFH-DA) (CAS No. 4091-99-0) ≥ 97%, hydrogen peroxide solution 30% (w/w) in H_2_O (CAS No. 7722-84-1), peroxidase from horseradish Type VI-A (CAS No. 9003-99-0) ≥ 250 U/mg solid, 4,6-diamidine-2-phenylindole (DAPI) (CAS No. 28718-90-3) and 6-hydroxy-2,5,7,8-tetramethylchromane-2-carboxylic acid (Trolox) 97% (CAS No. 53188-07-1) were purchased from Sigma-Aldrich Co. Titanium dioxide nanoparticles (TiO_2_ NP; rutile:anatase 15:85) (CAS No. 13463-67-7, size 25 nm) (Degussa-Evonik). Cell culture media components were all Gibco products and purchased from Thermo Fisher Scientific Inc. Formamidopyrimidine DNA glycosylase (fpg) (New England Biolabs Inc).

### Nanoparticle preparation and characterization

CeO_2_ NP was suspended in SH-SY5Y and A172 culture media (composition described below) at 100 µg/mL in both cases and ultrasonicated as previously described (Fernández-Bertólez et al. 2025). Serial dilutions were made to achieve concentrations ranging from 1 to 100 µg/mL (corresponding to 0.30–30.30 µg/cm^2^). Average hydrodynamic size, polydispersity index (PdI) and zeta potential of NP suspensions were assessed by dynamic light scattering (DLS) and mixed mode measurement phase analysis light scattering (M3-PALS), respectively, using a Zetasizer Nano-ZS equipped with 4.0 mW, 633 nm laser (Model ZEN 3600; Malvern Instruments Ltd., Malvern, Worcestershire, UK). The supplier reported a primary particle size of < 25 nm for cerium (IV) oxide nanopowder, determined by Brunauer–Emmett–Teller specific surface area analysis.

### Cell cultures

Human glioblastoma A172 (ECACC 88062428) and neuroblastoma SH-SY5Y (ECACC 94030304) cell lines were purchased from the European Collection of Authenticated Cell Cultures. Cultures were stablished and maintained in stable conditions and optimum growing rate for successive testing. A172 cells were cultured in a nutrient mixture of DMEM (high glucose), 2 mM l-glutamine, 1% antibiotic and antimycotic solution, and 10% heat-inactivated fetal bovine serum (FBS). Culture medium for SH-SY5Y cell line consisted of DMEM/F12 (1:1) medium, supplemented with 10% FBS, 1% non-essential amino acids, and 1% antibiotic and antimycotic solution. Cells were incubated with 5% CO_2_ at 37 °C in a humidified atmosphere.

### Cellular uptake of nanoparticles

Prior to carrying out the toxicity assessment in vitro, the internalization rates of CeO_2_ NP by the SH-SY5Y neuronal and A172 glial cells were assessed by flow cytometry methodology according to the protocol described by Fernández-Bertólez et al. (2024). Briefly, both cell types were seeded independently (5 × 10^4^ cells/well) in 96-well plates and treated with a wide range of CeO_2_ NP doses (1, 2, 5, 10, 25, 50 and 100 µg/mL, corresponding to 0.30, 0.61, 1.52, 3.03, 7.58, 15.15, and 30.30 µg/cm^2^) for three exposure periods (3, 24 and 48 h) at 37 ºC. Negative and positive controls (specific culture medium without CeO_2_ NP and 200 µg/mL TiO_2_ NP, respectively) were included in each experiment. Nanoceria and TiO_2_ NP suspensions were previously ultrasonicated to ensure the correct dispersion of particles as described above. After treatments, assessments were conducted in a FACSCalibur flow cytometer (Becton Dickinson S.A.) as previously described (Fernández-Bertólez et al. 2024), and the percentage of cells that incorporated CeO_2_ NP was calculated using the Cell Quest Pro software (Becton Dickinson). Three independent experiments were performed, and each condition was tested in duplicate.

### MTT assay

Cells (2 × 10^4^) were seeded in 96-well plates and allowed to adhere overnight. Then, cells were treated for 3, 24, and 48 h with different CeO_2_ NP concentrations (1, 2, 5, 10, 25, 50, and 100 µg/mL), or with a negative or positive control (specific culture medium without NP or 1% Triton X-100, respectively). MTT assay was carried out according to Mosmann (1983) with some modifications (Valdiglesias et al. 2023) to ensure that CeO_2_ NP did not interfere with the reagents used (light absorption interference) or with the detection method (catalytic interference) (Bessa et al. 2017). At least three independent experiments were carried out and each experimental condition was performed in triplicate.

### Morphological analysis

Changes in the normal shape of SH-SY5Y and A172 glial cells exposed to 1, 10 and 100 µg/mL CeO_2_ NP for 3 and 24 h were explored microscopically. Particularly, after treatments, cells were visualized under a light microscope (Nikon TMS, Nikon Corporation, Tokyo, Japan), and phase contrast photographs of control (not treated cells) and exposed cells were acquired to perform a qualitative analysis of cell morphology at all experimental points. Photographs were then processed using the ImageJ program (version 1.54 k, 2024).

### ***Acellular ROS generation by CeO***_***2***_*** nanoparticles***

The intrinsic cell-free ROS formation potential of CeO_2_ NP was assessed using the 2′,7′-dichlorodihydrofluorescein diacetate (DCFH-DA) assay, following the method previously described by Foucaud et al. (2007), with some modifications. Briefly, 20 mM DCFH-DA (in DMSO) was diluted to a concentration of 2 mM in 0.01 M NaOH and left for 30 min (at room temperature in the dark) to chemically hydrolyze the probe at basic pH. Afterward, 0.1 M PBS (pH 7.4) was added to stop the reaction, giving a final concentration of 20 µM 2′,7′-dichlorodihydrofluorescein (DCFH) (reaction mix). The solution was prepared just before use and placed on ice. Also, a 20 µM DCFH-DA solution (in DMSO) was prepared to be employed as blank.

The reaction mix solution was transferred to a black flat bottom 96-well plate (225 μL/well). Thereafter, 25 μL of PBS (negative control) or CeO_2_ NP suspensions (in PBS) was added to each well to give final concentrations of 1, 10 or 100 μg/mL. In addition, a negative control (reaction mix without NP) and a positive control (reaction mix plus 1 mM H_2_O_2_) were tested. In the wells containing H_2_O_2_, the enzyme horseradish peroxidase was added at 0.04 U/mL to catalyze generation of ·OH radicals since H_2_O_2_ cannot oxidize DCFH by itself (Pal et al. 2012). Afterward, the plate was promptly placed on ice, and fluorescence from the oxidation of DCFH to 2′,7′-dichlorofluorescein (DCF) was measured at 485/530 nm (excitation/emission) using a VICTOR Nivo™ multimode plate reader (PerkinElmer Inc., Massachusetts, USA) at time zero. Data were expressed as percentage of ROS regarding the negative control. All conditions were analyzed in triplicate in a minimum of three independent experiments.

Prior to conducting DCFH-DA assay, CeO_2_ NP autofluorescence at the excitation/emission wavelengths used in the assay was discarded.

### ***Intracellular ROS generation by CeO***_***2***_*** nanoparticles***

The cellular-mediated generation of ROS after CeO_2_ NP exposure was also measured by DCFH-DA assay. Cells were seeded in black flat bottom 96-well plates (5 × 10^4^ cells/well) and incubated overnight. On the following day, cells were exposed to different concentrations of CeO_2_ NP for 3 and 24 h. Cells without NP and cells treated with 1 mM H_2_O_2_ for 15 min were considered as negative and positive control, respectively. Cells were then washed twice with PBS and incubated with 20 μM DCFH-DA in PBS for 1 h at 37 °C. They were further analyzed by spectrofluorimetry as described above (previous section). A minimum of three independent experiments were carried out and each experimental condition was analyzed in triplicate. Data were expressed as percentage of ROS regarding the negative control.

### ***Intracellular scavenging capacity of CeO***_***2***_*** nanoparticles***

The ability of CeO_2_ NP to scavenge, neutralize, or mitigate ROS produced by an oxidant agent (H_2_O_2_) was also evaluated by DCFH-DA assay, thus providing insights into their antioxidant properties. To that aim, SH-SY5Y or A172 cells were seeded at 5 × 10^4^ cells/well in black flat bottom 96-well plates and allowed to adhere for 24 h. Afterward, cells were first exposed for 15 min to 1 mM H_2_O_2_ to produce ROS in a controlled manner. Then cells were washed with PBS and incubated with CeO_2_ NP (1, 10 and 100 μg/mL) in serum-free culture media for 3 and 24 h. Cells initially treated with 1 mM H_2_O_2_ with no further NP exposure and incubated for 3 and 24 h served as a control for intrinsic basal ROS presence (basal control). Cells treated only with H_2_O_2_ and immediately analyzed served as a positive control for the maximum ROS amount generated in the cellular system. Additionally, to serve as a ROS scavenging positive control, cells were treated with 1 mM H_2_O_2_ and subsequently with a known antioxidant substance (0.075 mM Trolox) for 3 and 24 h. At the end of exposure, cells were washed twice with PBS and incubated in the presence of 20 μM DCFH-DA in PBS for 1 h at 37 °C in the dark. Thereafter, plates were quickly placed on ice away from light, and fluorescence intensity was measured as described in acellular ROS quantification section. A minimum of three independent experiments were performed and each experimental condition was analyzed in triplicate. Data are expressed as percentage of ROS regarding H_2_O_2_-treated control cells.

### Fpg-modified comet assay

To assess oxidative DNA damage induced by CeO_2_ NP in SH-SY5Y and A172 cells, the fpg-modified comet assay was conducted. The methodology followed adhered to the Minimum Information for Reporting Comet Assay procedures and results (MIRCA) guidelines (Møller et al. 2020).

For each experimental condition, duplicate slides were prepared, with two agarose drops per slide. Following CeO_2_ NP treatments, cells were collected, and 20 µL of the remaining cell suspension was mixed with 80 µL of 0.9% low-melting-point (LMP) agarose, achieving a final concentration of 0.72%. The slides, previously coated with a 1% normal-melting-point agarose layer, were prepared adding two drops of 40 µL each of cellular agarose suspension, covered with coverslips (20 × 20 mm) and placing on ice for 15 min. Afterward, slides were immersed, without coverslips, overnight in lysis solution (2.5 M NaCl, 10 mM Tris–HCl, 250 mM NaOH, 100 mM Na_2_EDTA, pH 10, and 1% Triton X-100 added just before use) at 4 °C. Slides were washed 3 times (5 min each) with enzyme buffer (0.5 mM EDTA, 0.1 M KCl, 0.2 mg/mL bovine serum albumin, 40 mM HEPES, pH 8.0). One duplicate was treated with 50 µL fpg enzyme (0.0015 U/µL buffer), and the other one with 50 µL of enzyme buffer. All slides were then located in a humidified box and incubated at 37 ºC for 30 min. Subsequently, standard alkaline comet assay was carried out as described by Fernández-Bertólez et al. (2024).

DNA damage was quantified based on the percentage of DNA present in the comet tail (%tDNA). The oxidative DNA lesions detected were expressed as net fpg-sensitive sites, calculated by subtracting the %tDNA obtained from the buffer-incubated slide from that of the enzyme-incubated one for each condition. To account for inter-experimental variability, SH-SY5Y cells exposed to 1.5 mM potassium bromate were included in all experiments as positive reference standards, and data normalization was performed in accordance with the approach outlined by Collins et al. (2014).

Prior to conducting the fpg-modified comet assay, potential interference of CeO_2_ NP with fpg enzyme activity was evaluated following the methodology described by Magdolenova et al. (2012). In brief, SH-SY5Y and A172 cells were independently incubated for 1 h either with 1.5 mM KBrO_3_ plus CeO_2_ NP at the highest concentration tested (100 µg/mL), or just with KBrO_3_. Then, fpg-modified comet assay was carried out as described above.

### Statistical analysis

SPSS for Windows statistical package (version 29.0) was used to perform the statistical analyses. In particular, non-parametric tests Mann–Whitney *U* test and Kruskal–Wallis test were used to analyze differences among groups and to carry out two by two comparisons, respectively. Associations between two variables were explored by means of Pearson`s correlation. Experimental data were expressed as mean ± standard error and a *p* value of 0.05 was considered significant.

## Results

In this study, human SH-SY5Y and glial A172 cells were treated with CeO_2_ NP and a series of outcomes related to cell morphology and viability, and to their ability to modify oxidative species production or to induce oxidative DNA damage was investigated.

### Nanoparticle characterization

Table 1 summarizes the main physicochemical characteristics of CeO_2_ NP suspended in cell culture media at the highest concentration used for toxicity assessment (100 µg/mL). Results of DLS evaluation revealed that CeO_2_ NP remained stable without agglomeration in both SH-SY5Y and A172 cell culture media since their hydrodynamic size maintained with minimal fluctuations throughout all times tested. Furthermore, PdI values obtained around 0.3 indicate a homogeneous and mostly monodisperse nanoparticle population. Zeta potential values were negative and notably stable at all conditions tested.

**Table 1:** Physicochemical characterization of CeO_2_ nanoparticles

Dispersion medium	Time point (h)	Hydrodynamic diameter (nm)	PdI	Zeta potential (mV)
A172 culture medium	0	140.1 ± 3.0	0.26 ± 0.01	− 11.8 ± 0.7
	3	142.0 ± 3.6	0.25 ± 0.01	− 12.1 ± 0.7
	24	156.2 ± 7.7	0.27 ± 0.07	− 12.6 ± 0.7
	48	166.7 ± 6.5	0.18 ± 0.04	− 12.4 ± 0.7
SH-SY5Y culture medium	0	148.1 ± 19.8	0.31 ± 0.04	− 11.3 ± 0.9
	3	134.4 ± 4.9	0.29 ± 0.01	− 11.5 ± 0.5
	24	139.5 ± 3.7	0.30 ± 0.01	− 11.3 ± 1.1
	48	139.3 ± 3.5	0.30 ± 0.03	− 11.7 ± 0.7

### ***Cellular uptake of CeO***_***2***_*** nanoparticles***

**Fig. 2:**
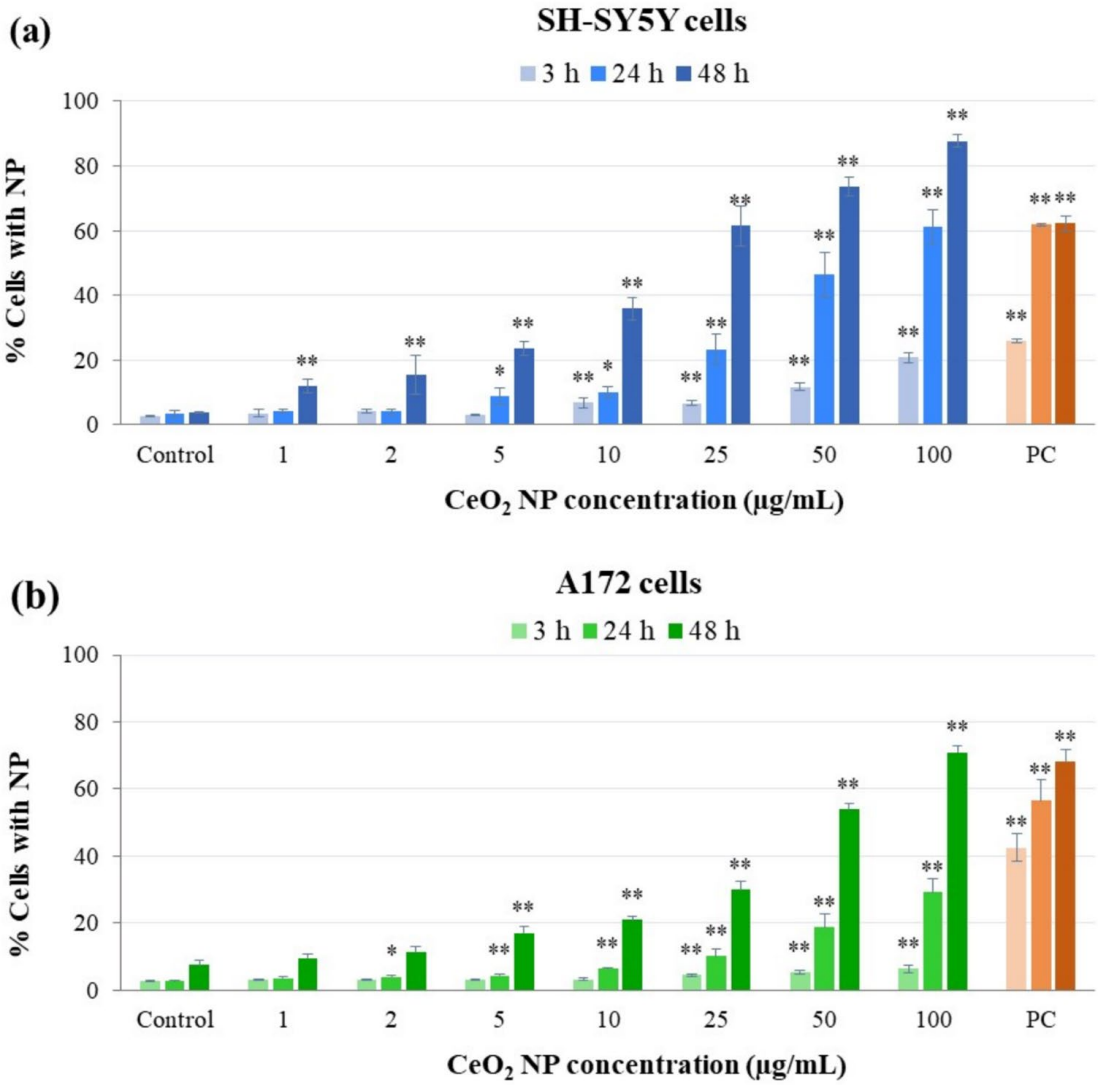
Flow cytometry analysis of cellular uptake in SH-SY5Y neurons **a** and A172 glial cells **b** exposed to CeO_2_ NP for 3, 24 and 48 h. * *p* < 0.05, ** *p* < 0.01, significant differences regarding the corresponding control. PC, positive control (200 µg/mL TiO_2_ NP)

Flow cytometry analysis of CeO_2_ NP internalization by SH-SY5Y and A172 cells (Fig. 2) revealed a similar dose- and time-dependent NP uptake in both cell types, being the uptake rate slightly higher in neurons than in glial cells. Specifically, significant increases were observed in SH-SY5Y cells from 10 μg/mL onwards at 3 h, from 5 μg/mL onwards at 24 h, and at all concentrations tested at 48 h (concentration–response relationships: *r* = 0.789, *p* < 0.01 at 3 h; *r* = 0.854, *p* < 0.01 at 24 h; and *r* = 0.935, *p* < 0.01 at 48 h). A172 cells efficiently took CeO_2_ NP up from 25 μg/mL onwards after 3 h treatment, from 2 μg/mL onwards at 24 h, and from 5 μg/mL onwards at 48 h of exposure (concentration–response relationships: *r* = 0.559, *p* < 0.01 at 3 h; *r* = 0.760, *p* < 0.01 at 24 h; and r = 0.919, *p* < 0.01 at 48 h).

### Effect on cell viability

**Fig. 3:**
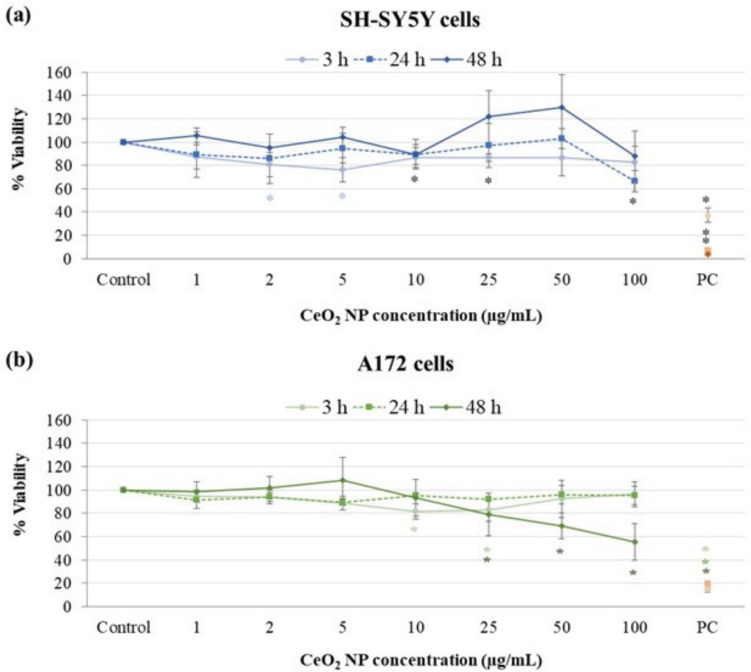
Cellular viability assessment of SH-SY5Y neuronal cells **a**, and A172 glial cells **b** exposed to CeO_2_ NP for 3, 24 and 48 h. **p < 0.01, significant differences regarding the corresponding control. PC, positive control (1% Triton X-100)

Figure 3a shows the results of the MTT test, modified to avoid any interference of the CeO_2_ NP with the assay reagents of detection method, in neuronal cells. Data revealed low cytotoxicity across all time points tested, generally maintaining viability values above 80%. According to the criterion established by ISO 10993-5 (ISO 2009), the reduction in cell viability that did not exceed 30% is not considered as a cytotoxic effect since is not biologically relevant. Therefore, we can consider CeO_2_ NP did not induce cytotoxic effects in SH-SY5Y cell line. Similarly, Fig. 3b shows that these NP did not exhibit significant cytotoxic effects in A172 cells either as cell viability remained above 80% after shorter exposure times (3 and 24 h). After 48 h exposure, a progressive decline in cell viability from 25 µg/mL onwards was observed, indicating a cytotoxic activity of these NP in glial cells under these more extreme conditions.

Half-maximal inhibitory concentration (IC_50_) was calculated from the MTT results for each cell line and exposure time. IC_50_ was not reached in any of the cell types exposed for 3 h, indicative of the low anti-proliferative potential of these NP in short-term exposure periods. Values obtained for 24 and 48 h were consistently high (above 100 μg/mL), very similar in neuronal cells (114.42 ± 6.96 for 24 h, and 116.11 ± 9.58 for 48 h), and showing a progressive decline with time in glial cells (240.35 ± 4.89 for 24 h, and 104.61 ± 33.68 for 48 h).

### Morphological analysis

**Fig. 4:**
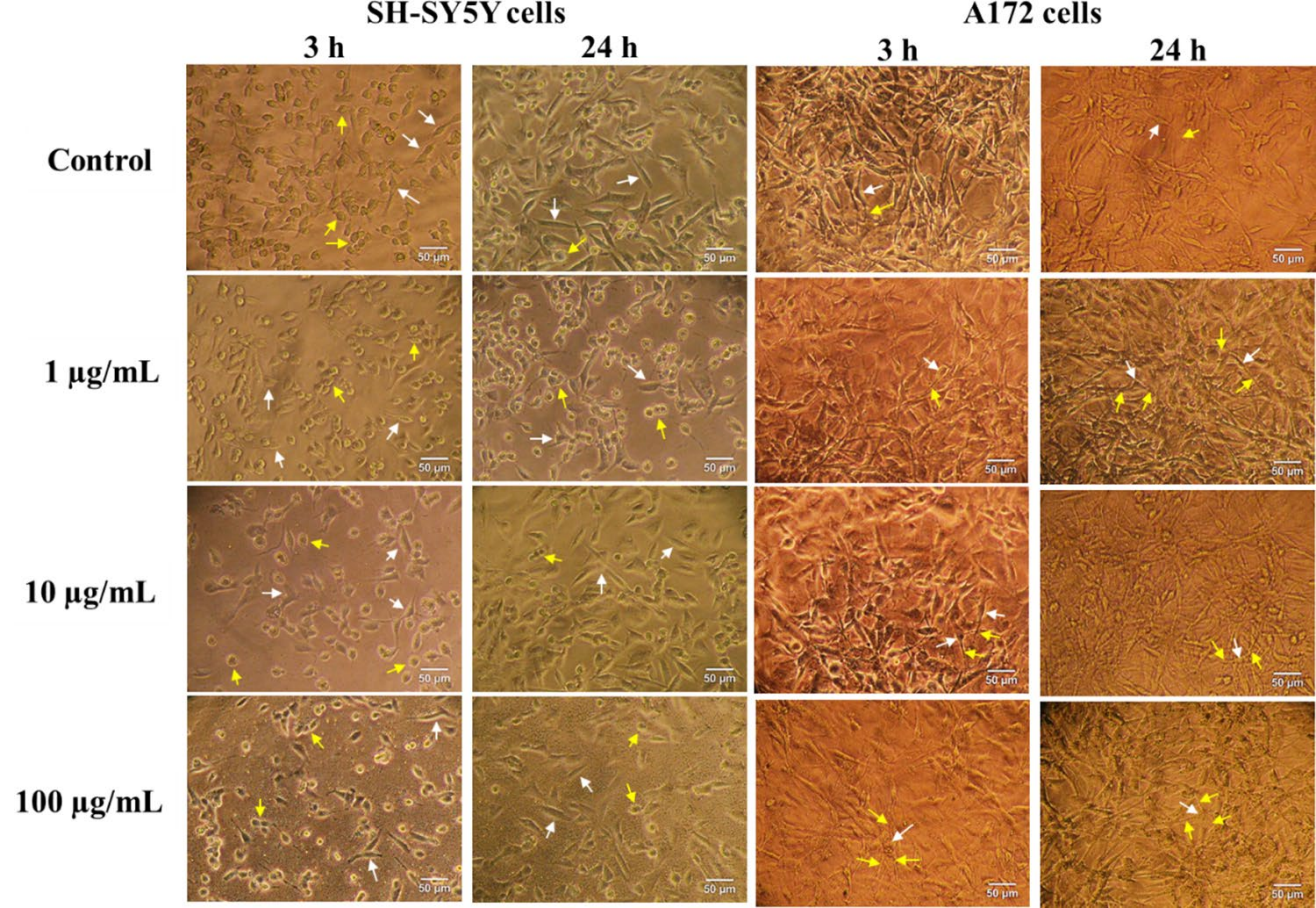
Cytomorphological analysis of undifferentiated SH-SY5Y cells (left panels) and A172 cells (right panels), untreated and exposed to different concentrations of CeO_2_ NP for 3 and 24 h. In SH-SY5Y cells, white arrows point at neuronal cells with spindle-like morphology with few dendritic cytoplasmic protrusions (processes), and yellow arrows indicate cells with epithelial-like morphology. In A172 cells, **w**hite arrows point at the body of polygonal-shaped to fibroblast-like cells, and yellow arrows indicate the characteristic and numerous processes of the astrocytes, a subtype of glial cells

Morphology of SH-SY5Y cells, untreated and exposed to CeO_2_ NP, is depicted in Fig. 4. Control undifferentiated cells tend to grow in clusters formed mainly by round or oval cells on top of each other (yellow arrows), mixed with polarized neuroblast-like cell bodies with few and incipient truncated short processes (white arrows). Only after treatment with the highest concentration of CeO_2_ NP (100 µg/mL), a decrease in density and compaction of cell clusters was observed, along with a slight decrease in the proportion of cells showing the neuroblast-like morphology, and in the size and number of their incipient neurites.

Figure 4 also displays morphological analysis of A172 glial cells. They exhibit distinctive morphological characteristics typical of astrocytic cells, consisting of giant fibroblast-like cells (white arrows) with numerous processes extending from the cell body (yellow arrows). A172 cells can display a multiform morphology in culture, with spindle‐form or polygonal to amorphous shapes, and grow as strongly adhered monolayer or in foci without evidence of contact inhibition. Exposure to CeO_2_ NP did not induce changes regarding control cells in either cell density or the typical cell morphology at any of the conditions tested.

### ***Acellular ROS generation by CeO***_***2***_*** nanoparticles***

**Fig. 5:**
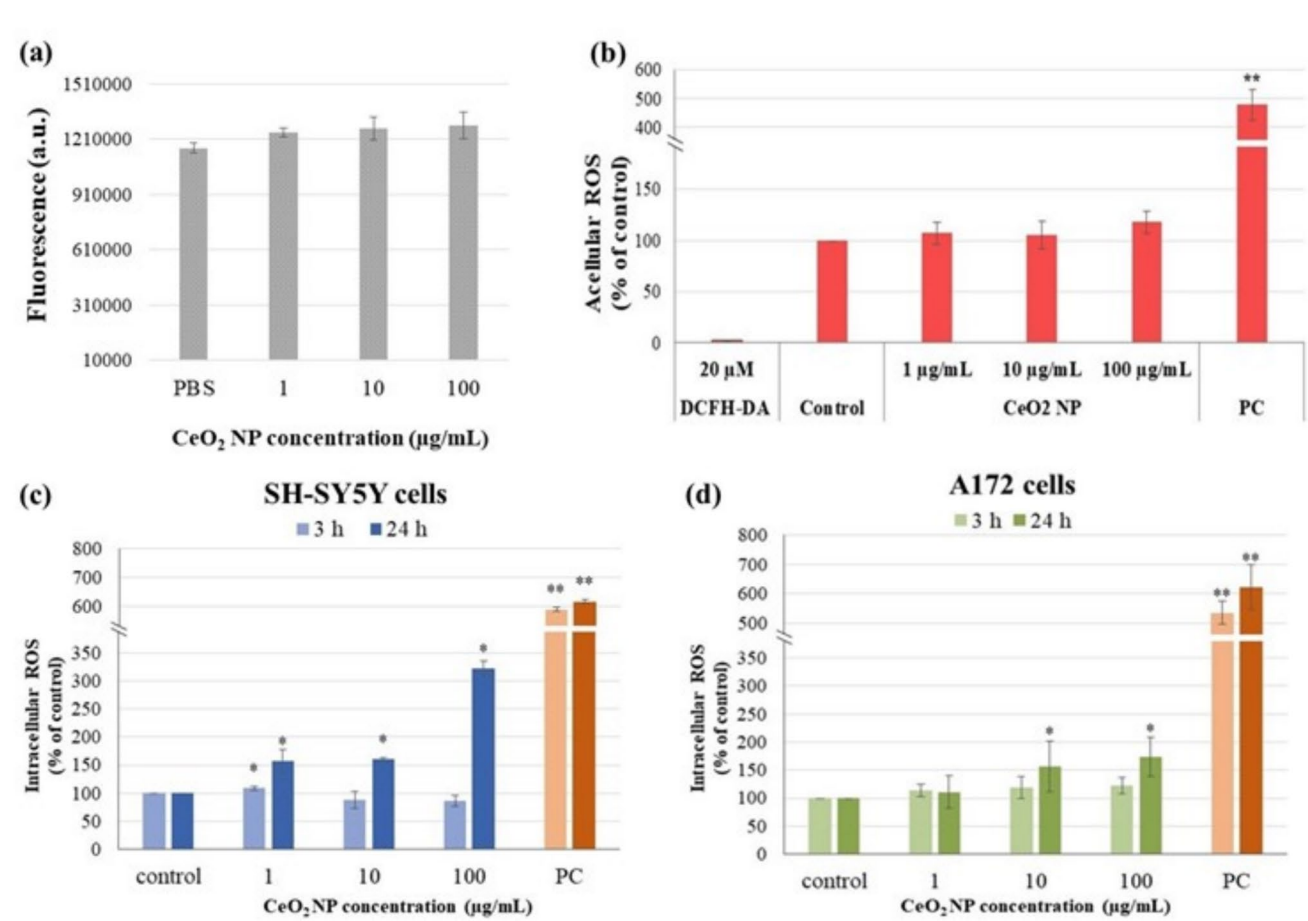
Reactive oxygen species (ROS) generation by CeO_2_ NP. Autofluorescence of CeO_2_ NP at 485/530 nm (excitation/emission) **a** acellular ROS production **b** intracellular ROS generation in SH-SY5Y neuronal cells **c** and in A172 glial cells **d**. *p < 0.05, **p < 0.01, significant differences regarding the corresponding control. a.u., absorbance units; PBS, phosphate-buffered saline; DCFH-DA, 2′,7′-dichlorodihydrofluorescein diacetate; PC, positive control (1 mM H_2_O_2_ + 0.04 U/mL horseradish peroxidase for acellular assay, and 1 mM H_2_O_2_ for intracellular assay)

Prior to conducting acellular and cellular DCFH-DA assay, autofluorescence of CeO_2_ NP at the same excitation/emission wavelengths used in the assay was tested. As it is shown in Fig. 5a, fluorescence of the CeO_2_ NP suspensions at all concentrations was not higher than the one of PBS.

Results of the evaluation of CeO_2_ NP pro-oxidant ability in a cell-free system are gathered Fig. 5b. None of the concentrations tested was capable of oxidizing the DCFH per se under the conditions of the present work as shown by the absence of significant differences in fluorescence intensity compared to the control.

### ***Intracellular ROS generation by CeO***_***2***_*** nanoparticles***

DCFH-DA assay was also employed to test the intracellular ROS levels generated by CeO_2_ NP in the presence of neuronal and glial cells (Fig. 5c and d). After exposing SH-SY5Y cells to nanoceria for 3 h, no significant ROS production was observed at any of the concentrations tested. On the contrary, after 24 h treatments, a significant concentration-dependent increase in the amount of ROS was obtained (*r* = 0.884; *p* < 0.01), reaching a threefold increase with regard to control cells at the highest concentration (100 μg/mL).

Similarly, A172 cells exposed for 3 h to CeO_2_ NP showed no effect on ROS production, and those treated for 24 h experienced a slight although significant increase in the intracellular ROS levels at 10 and 100 μg/mL, with no significant concentration–response relationship.

### ***Intracellular scavenging capacity of CeO***_***2***_*** nanoparticles***

**Fig. 6:**
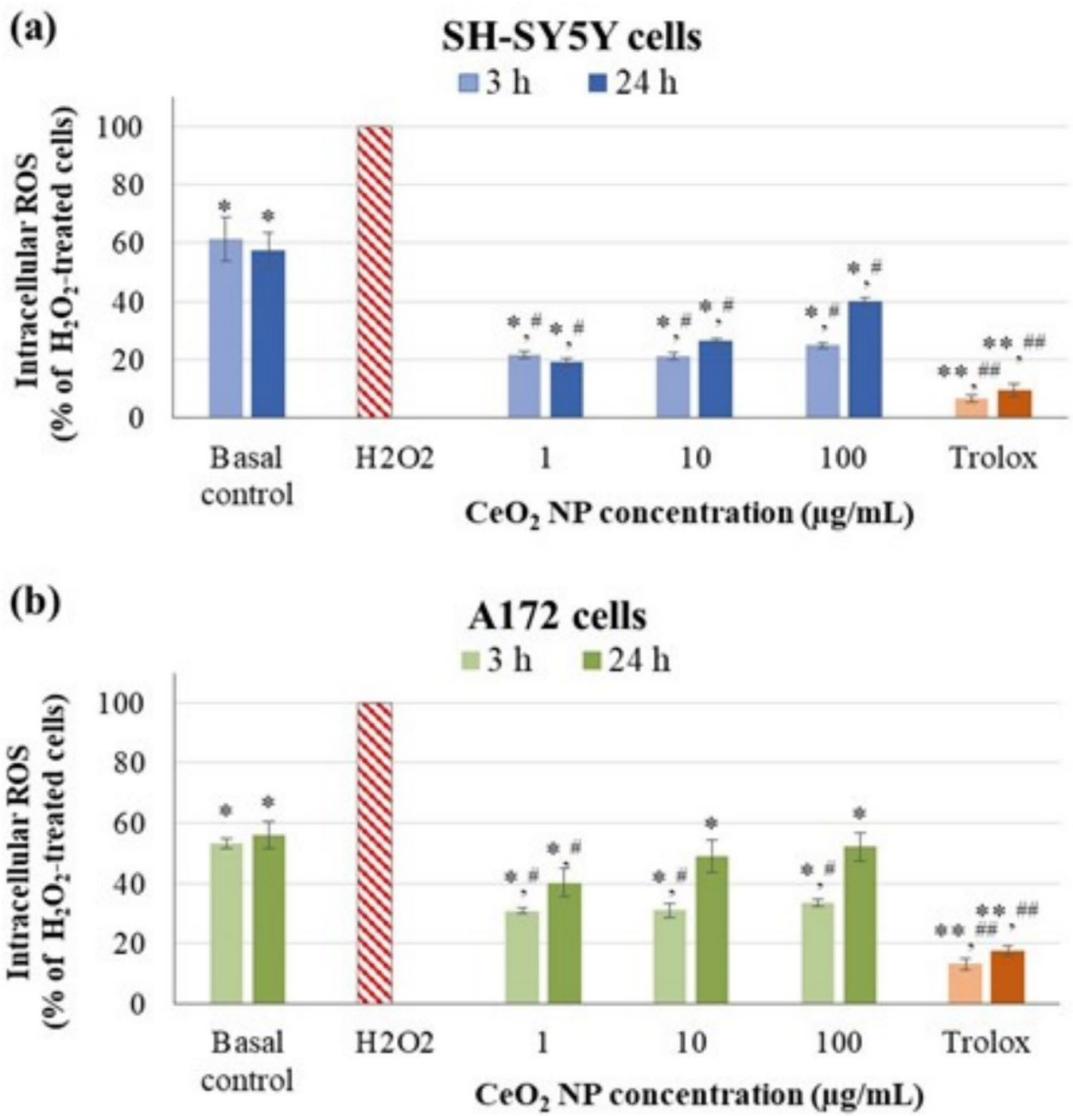
Intracellular scavenging capacity of CeO_2_ NP in SH-SY5Y cells a, and A172 cells b. *p < 0.05, **p < 0.01, significant differences regarding H_2_O_2_-treated cells at time = 0 h (striped red bar). ^##^*p* < 0.01, ^#^*p* < 0.05, significant differences regarding the basal control

The ability of CeO_2_ NP to scavenge ROS generated by a known oxidant (H_2_O_2_) in neuronal and glial cells was also elucidated by means of DCFH-DA assay (Fig. 6). Nanoceria, regardless of the concentration and after both 3 and 24 h of incubation, was effective in reducing H_2_O_2_-induced intracellular ROS in SH-SY5Y cells (around 80% at 3 h, and between 60 and 82% at 24 h) and in A172 cells (around 70% for 3 h and between 48 and 60% at 24 h). Their effectiveness was more pronounced in neuronal cells than in glial cells, and higher than the intrinsic ability of both cell types to reduce H_2_O_2_-induced ROS during the same incubation periods (basal controls).

### Oxidative DNA damage evaluation

**Fig. 7:**
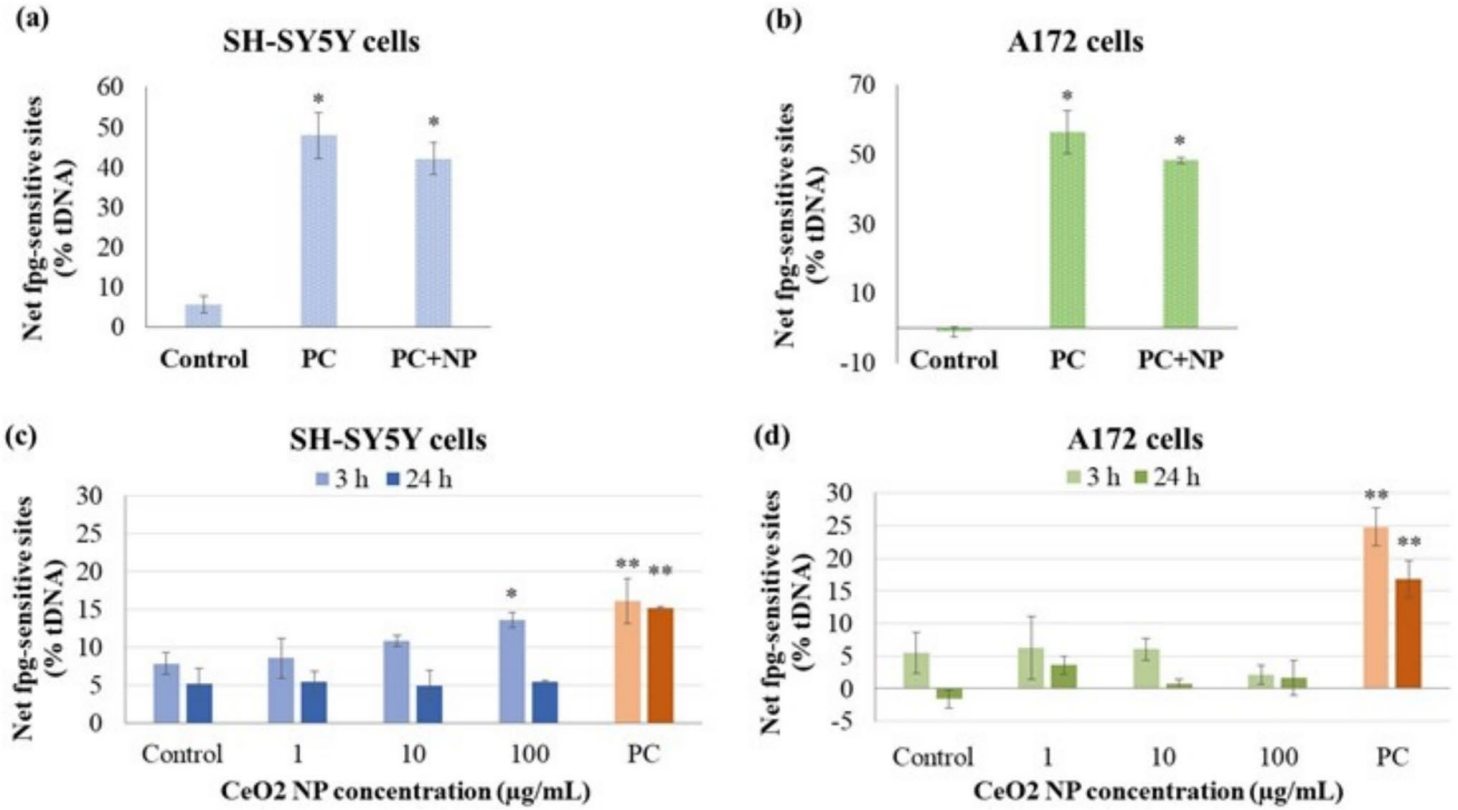
Results of fpg-modified comet assay. Interference of CeO_2_ NP (100 µg/mL) with fpg enzyme in SH-SY5Y neuronal cells **a**, and A172 glial cells **b**, and oxidative DNA damage induced by CeO_2_ NP in SH-SY5Y cells **c** and A172 cells **d** *p < 0.05, **p < 0.01, significant difference regarding the corresponding control. PC: positive control (1.5 mM KBrO_3_)

Before evaluating the potential oxidative DNA damage caused by nanoceria by means of the fpg-modified comet assay, the possible interference of these NP with fpg enzyme activity was evaluated. Figure 7a and b show that the presence of CeO_2_ NP did not interfere with the detection of oxidative DNA lesions induced by a known oxidant (KBrO_3_) in any of the cell types studied since fpg enzyme efficiently detects oxidative DNA damage regardless the presence of NP.

Data obtained in the fpg-modified comet assay in SH-SY5Y cells showed that significantly increased oxidative DNA damage was just obtained at the CeO_2_ NP highest concentration tested after 3 h of exposure, while no evident effects were observed after 24 h (Fig. 7c). CeO_2_ NP exposure did not induce oxidative damage in the DNA of A172 cells under any of the conditions tested (Fig. 7d).

## Discussion

This study addressed the potential ROS-mediated cellular effects of CeO_2_ NP, as well as their capacity to scavenge ROS, focusing on human nervous system cells. Results of characterization analysis revealed that CeO_2_ NP were stable and did not agglomerate in either SH-SY5Y or A172 culture media since their average hydrodynamic diameter and zeta potential maintained with minimal variations throughout all times tested. Besides, PdI results showed that the size distribution is homogeneous and mostly monodisperse even up to 48 h. These results are consistent with those described by Kumari et al. (2014) in IMR32 neuronal cell culture medium (similar to SH-SY5Y medium) for the same NP used in the present study, by Patel et al. (2018) for self-synthesized CeO_2_ NP in water, and by Rodea-Palomares et al. (2011) in water.

Prior to toxicity testing, internalization of CeO_2_ NP into SH-SY5Y and A172 cells was evaluated by flow cytometry, considered as a rapid and sensitive technique for quantitative determining the cellular NP uptake (Ibuki and Toyooka 2012; Ostermann et al. 2020). Results obtained showed that both nervous cell types were able to efficiently internalize CeO_2_ NP in a dose- and time-dependent manner, although the uptake rate was slightly higher in SH-SY5Y cells than in A172 ones. These results align with other previous studies that demonstrated, through various techniques, the effective internalization of CeO_2_ NP in different human cell types, including A549 lung cells (Diaconeasa et al. 2015), HeLa cervical cancer cells and HFL-1 fetal lung fibroblasts (Diaconeasa et al. 2017), BEAS-2B cells (Ballesteros et al. 2021), and U937 monocytes (Lord et al. 2012; Ting et al. 2013). Furthermore, previous studies have shown that the uptake of nanoceria by HaCat cells is carried out mainly by endocytic pathways, and could be co-localized with lysosomes, endoplasmic reticulum and mitochondria, in addition to being abundant in the cytoplasm and nucleus (Singh et al. 2010); however, the flow cytometry methodology employed in the present study did not allow to determine the mechanism of internalization, and further research is required to elucidate the exact uptake mechanism in neuronal and glial cells.

In order to evaluate CeO_2_ NP effect on cell viability, a modified version of MTT assay avoiding NP interference was carried out (Bessa et al. 2017; Valdiglesias et al. 2023). Absence of cytotoxicity of CeO_2_ NP was observed for most conditions tested since cell viability values were above 80% in all cases, except in A172 cells exposed for 48 h at the highest concentrations. Results obtained in previous studies carried out in cells from neural origin revealed, in general, significant dose and time-dependent decreases in cell viability although cytotoxicity (> 30% reduction in viability) was rarely reached. Specifically, human IMR32 neuroblastoma cells treated with CeO_2_ NP for 24 h showed significant decreases in cell viability at the highest concentrations tested (50, 100, and 200 μg/mL) though this parameter remained above 70% in all cases (Kumari et al. 2014). Likewise, D’Angelo et al. (2009) demonstrated that nanoceria did not affect SH-SY5Y cell viability up to a concentration of 100 μg/mL, while higher concentrations (150 and 200 μg/mL) induced a significant decrease in viability after 24 h of exposure, only below 70% at the highest one. And exposure of HT-22 mouse hippocampal neurons to CeO_2_ NP (0.0002–20 µg/mL) induced very little toxicity, showing a high biocompatibility (Schubert et al. 2006). Even exposure to these NP at notably higher concentrations did not affect cell viability; this was the case of undifferentiated and differentiated neuronal SH-SY5Y cells exposed to 24 h with polyacrylic acid-CeO_2_ NP (0.75–3 mM ≈ 0.9–3.5 mg/mL) (Meenambal et al. 2023) and of BV-2 mouse microglial cells exposed to nanoceria at 100 µg/mL for 24 and 48 h (Sikorska et al. 2020).

Results reported in cells from non-neural origin were in the same line. Thus, human A375 skin melanoma cells were treated with the same CeO_2_ NP employed in our study under similar experimental conditions (20–120 µg/mL for 24 and 48 h), and viability showed concentration and time-dependent decreases, remaining above 70% in almost all cases (Ali et al. 2015). Similarly, viability of A549 lung cells treated with nanoceria decreased in a concentration (3.5–23.3 μg/mL) and time (24–72 h)-dependent manner although only the highest concentration surpassed the 30% cytotoxicity threshold (Lin et al. 2006). A high biocompatibility was observed in the same cell line treated with 1 to 500 μg/mL CeO_2_ for 24 h, indicated by an IC_50_ > 500 µg/mL (Diaconeasa et al. 2015), in THP-1 cells exposed for 24 h to 10–100 μg/mL (Patel et al. 2018), and in human U937 monocytic cells treated with 5 and 200 μg/mL since the reduction in cell viability did not exceed 30% at 24 h, with no further changes until 120 h (Lord et al. 2012).

Based on all these studies and according to our results, it appears that the adverse cellular effects caused by CeO_2_ NP on cell viability are generally limited, primarily depending on the cell type and exposure time, and restricted to high NP doses. However, it is worth mentioning that just a few previous studies evaluated the potential interference of CeO_2_ NP with both reagents and detection systems of the viability assays employed (Guan et al. 2016; Meenambal et al. 2023). Hence, reliability of the results obtained is uncertain since interference may lead to over- or underestimation of viability (false positive or negative results).

Toxic agents may cause significant physiological and pathological changes in cells, including alterations in cell morphology, such as rounding, opacity, and detachment, often indicative of the triggering of different cell death processes (Hu et al. 2021). Thus, qualitative evaluation of changes in the appearance or typical shape of SH-SY5Y and A172 cells employed in the present study provided indirect information on the effect of CeO_2_ NP on cytoskeleton and plasma membrane integrity. In addition, alterations in the morphology of these cells would compromise the integrity and functionality of the CNS, contributing to enhance neurodegenerative processes, blood-brain barrier disruption, inflammation, and cognitive dysfunction (Martinelli et al. 2020). Only exposure to the highest concentration of CeO_2_ NP induced a slight decrease in the density of SH-SY5Y cells and their adhesion capacity. Furthermore, after the longer exposure period to these NP, the proportion of cells with spindle morphology and the number and complexity of their dendrites were also reduced. Furthermore, no alterations in the typical astrocyte morphology were observed after exposure. These results demonstrated that both nervous system cell types are able to maintain their typical morphology in the presence of CeO_2_ NP, which is crucial for the proper functioning of the nervous tissues.

These findings are consistent with others previously reported in the literature. Meenambal et al. (2023) observed no morphological changes in both undifferentiated and differentiated neuronal SH-SY5Y cells treated with 1.5 mM and 3 mM FITC labeled polyacrylic acid-CeO_2_ NP for up to 6 h; Peloi et al. (2020) described that nanoceria treatments (10 nM and 10 µM, equivalent to 0.02 and 1.72 μg/mL, respectively) helped preserve the typical morphology of UVB-irradiated L929 fibroblasts, maintaining their elongated and flattened appearance, with only a slight reduction in their filopodia; and Sarnatskaya et al. 2020) reported a low although progressive decrease of adhesive properties of MCF-7 breast cancer cells under influence of CeO_2_ NP (0.2–200 nM ≈ 0.000034–0.034 μg/mL). However, opposite to our results, the morphology of human skin melanoma A375 cells became altered into spherical shape and detached from surface after their exposure to nanoceria (the same employed in this study) from 80 µg/mL onwards for 24 and 48 h (Ali et al. 2015). Similarly, A549 cells became round at 25 µg/mL concentration. At higher doses, many cells detached and formed irregular clumps, with reduced cell density at 100 µg/mL (Mittal and Pandey 2014).

NP may cause cellular toxicity through different mechanisms. Among them, the induction of oxidative stress through excessive ROS production seems to be the most frequently suggested (Martinelli et al. 2020). Elevated ROS levels may harm cells by causing lipid peroxidation, mitochondrial damage, gene transcription modulation, DNA disruption, and protein oxidation, ultimately leading to cell apoptosis/death (Yaghoobi et al. 2020; Samrot and Noel Richard Prakash 2023). On this basis, in the present study, oxidative stress induced by CeO_2_ NP was investigated as a possible action mechanism for the effects previously reported in the nerve cells at cellular and genetic levels (Fernández-Bertólez et al. 2024). The study design included an initial evaluation of the intrinsic capacity of these NP to increase ROS production in the absence and in the presence of cells, analyzing the consequences of oxidative stress on genetic material, and exploring whether nanoceria has the capacity to eliminate or reduce ROS, acting as a potential antioxidant as previously suggested in the literature (Kwon et al. 2016; Nelson et al. 2016).

The possibility that CeO_2_ NP can produce ROS intrinsically was discarded with the acellular DCFH-DA assay. In contrast with these results, Xia et al. (2008) observed significant abiotic production of ROS from 8 nm CeO_2_ NP although these NP were smaller than the current ones (8 nm vs. ≤ 25 nm), which can translate into higher surface reactivity due to a higher surface to volume ratio. Besides, cubic NP were employed in that study instead of spherical as in ours, and it was previously reported that particle shape has a direct effect on NP cytotoxicity (Egbuna et al. 2021). Evaluation of cellular ROS production showed positive results only after 24 h CeO_2_ NP treatments, with a moderate dose-dependent effect in neuronal cells and a milder response in glial cells. Although dying cells can produce free radicals, cell death is not the main source of ROS in the presence of CeO_2_ NP. This is because the tested conditions did not reduce cell viability by more than 30%. Several previous works reported dose-dependent low levels of ROS generation from nanoceria in HT-22 nerve cells exposed to 20 µg/mL (Schubert et al. 2006); in IMR32 neuroblast cells treated with 100 and 200 µg/mL (Kumari et al. 2014); in MCF-7 breast cancer cells treated with 1 to 6 mg/mL (Sarnatskaya et al. 2020); in A375 skin melanoma cells (20 to 120 µg/mL) (Ali et al. 2015); in SKOV3 ovarian and WiDr colorectal cancer cells treated with 50 µg/mL (Vassie et al. 2017); or in Hela cervical cancer cells and HFL-1 normal fetal lung fibroblasts exposed to 5–250 µg/mL (Diaconeasa et al. 2017).

Antioxidants are natural substances that protect tissues and organs from damage caused by oxidative agents. Generally, the human body contains a well-developed antioxidant protective system. Oxidative stress occurs when oxidative insults exceed antioxidant defenses. At this point, external antioxidants are required to overcome this issue (Yang et al. 2019). Data obtained in the current study demonstrated that the CeO_2_ NP possess a certain intrinsic ability to scavenge ROS generated by H_2_O_2_, more pronounced in the presence of neuronal cells than in the presence of glial cells. Both cell types showed a similar intrinsic antioxidant ability, and treatment with nanoceria contributed to reduce ROS levels beyond their basal capacity. Longer exposure to CeO_2_ NP (24 vs. 3 h) or higher concentrations did not contribute to a higher decrease in oxidative stress, but the opposite, suggesting that the antioxidant capacity of nanoceria is dampened with increasing dose and exposure time. This is likely related to the significant intrinsic production of ROS by CeO_2_ NP shown under these more extreme conditions (100 µg/mL for 24 h), especially intense in neuronal cells, thus involving changes in the redox state of the NP surface that derive in promoting oxidant over antioxidant properties. Our results confirm the findings of numerous previous studies reporting excellent anti-ROS ability of nanoceria in different cell types of nervous and non-nervous origin, mainly at low doses (between 1 and 25 µg/mL) and short-term exposure periods, suggesting that nanoceria with a high Ce^3+^/Ce^4+^ ratio could show, in general, enhanced antioxidant capacity (Sadidi et al. 2020; Martinelli et al. 2020; Goujon et al. 2021; Lord et al. 2021).

ROS interaction with DNA can result in single-strand breaks (SSB) and double-strand breaks (DSB), leading to the accumulation of various cytotoxic and/or mutagenic lesions (Muruzabal et al. 2021). The comet assay is a widely used, efficient, straightforward, and sensitive method for detecting primary damage in DNA. This includes single-strand breaks (SSB), double-strand breaks (DSB), alkali-labile sites, and incomplete excision repair sites in individual cells (Collins et al. 2023). The comet assay, when incorporating an additional incubation step with various repair enzymes, enables the specific detection of different types of DNA lesions. Notably, the presence of 8-oxo-7,8-dihydro-2′-deoxyguanosine adducts—one of the most prevalent DNA lesions resulting from ROS and widely recognized as a biomarker for oxidative stress—can be identified using fpg (Azqueta et al. 2019). In this study, the induction of oxidative DNA damage by CeO_2_ NP was determined by the fpg-modified comet assay; before conducting the experiments, possible interference of the nanoceria with fpg enzyme activity were ruled out. Data analysis revealed a significant increase in oxidative DNA damage just in neuronal cells exposed for 3 h to the highest CeO_2_ NP concentration tested. Negative results were obtained for all other experimental conditions. Although CeO_2_ NP generated a significant amount of intracellular ROS after 24 h in both cell types analyzed, particularly in SH-SY5Y cells, our data indicate that they did not induce oxidative damage in the DNA. These results support previous ones obtained in different cell types. For instance, lower levels of oxidative damage were detected in differentiated Caco-2 cells after 24 h of CeO_2_ NP treatment (10, 25, and 100 μg/mL), suggesting an antioxidant effect of nanoceria under those conditions (Vila et al. 2018). Similarly, Rubio et al. (2016) used the fpg-modified comet assay to demonstrate the ROS quenching ability of CeO_2_ NP (2.5, 5, and 7.5 μg/mL for 24 h) in BEAS-2B lung epithelial cells exposed to KBrO_3_, which specifically induces oxidative DNA damage (Møller et al. 2023).

However, contrary to our results, some other previous works reported oxidative DNA damage induced by nanoceria exposure. Mittal and Pandey (2014) reported that 1 to 100 μg/mL CeO_2_ NP induced ROS-mediated DNA damage in A549 lung cells. Moreover, exposure of mouse follicular cells to concentrations of nanoceria up to 100 µg/mL and their accumulation in the zona pellucida prevented the protection of mature oocytes from DNA damage and oxidative stress (Courbiere et al. 2013). Similarly, mouse oocytes cultured in media containing CeO_2_ NP (< 0.01 µg/mL) during in vitro fertilization had a considerably reduced fertilization rate, probably due to genotoxicity and oxidative stress induced in gametes by these NP (Preaubert et al. 2016; Bisht et al. 2017). These works seem to indicate that the possible oxidative genotoxic damage induced by nanoceria were not only dependent on the NP physicochemical characteristics, such as concentration, shape, or size, but would also be conditioned by the sensitivity of the cell type assessed.

Despite the demonstrated antioxidant capacity of CeO_2_ NP, few studies addressed the potential of nanoceria to reduce oxidative stress markers, especially in nerve cells. Goujon et al. (2021) investigated the presence of 8-OHdG in bEnd.3 cells after treatment with glutamate, associated or not with nanoceria. CeO_2_ NP, bare and with two different coats, induced a significant decrease in glutamate-induced 8-OHdG levels after 4 h of incubation, while only the treatment with coated CeO_2_ NP was effective after 24 h, demonstrating that nanoceria have no effect per se on prevention of oxidative lesions.

## Conclusions

Most neurodegenerative diseases are associated with increased oxidative stress and neuroinflammation that compromise brain integrity and function. The prominent behavior of cerium oxide as a regenerative antioxidant justifies exploring their neuroprotective effects of against cell damage induced by oxidative stress as a promising tool for related pathological conditions, including neurodegenerative diseases. Results obtained from the present study demonstrated that exposure of human nervous system cells, SH-SY5Y and A172, to CeO_2_ NP does not induce significant decreases of cellular viability, relevant morphology alterations of neuronal or glial cells that might compromise their integrity or function, or intrinsic ROS cell-free production. Even though intracellular ROS was detected after nanoceria treatment, this was limited to the longest exposure time (24 h) and resulted particularly severe only for SH-SY5Y cells at the highest concentration employed (100 μg/mL). Still, increased ROS levels were not related to oxidative DNA damage induction since, in general, no increase of fpg-sensitive sites was observed. Furthermore, in the presence of CeO_2_ NP, both cell lines were efficient in scavenging ROS induced by H_2_O_2_ treatment, especially at short-term treatments, confirming the antioxidant ability of these NP previously suggested in the literature. These results confirmed not only good biocompatibility of nanoceria in human nervous system cells, but also their ability to efficiently scavenge externally induced ROS, supporting further exploring their potential use in the biomedical field, particularly, for those therapeutic and diagnostic applications related to the nervous system.

## Data Availability

Data will be made available on request.
